# RNA Sequencing of Human Peripheral Blood Cells Indicates Upregulation of Immune-Related Genes in Huntington's Disease

**DOI:** 10.3389/fneur.2020.573560

**Published:** 2020-11-27

**Authors:** Miguel A. Andrade-Navarro, Katja Mühlenberg, Eike J. Spruth, Nancy Mah, Adrián González-López, Tommaso Andreani, Jenny Russ, Matthew R. Huska, Enrique M. Muro, Jean-Fred Fontaine, Vyacheslav Amstislavskiy, Alexei Soldatov, Wilfried Nietfeld, Erich E. Wanker, Josef Priller

**Affiliations:** ^1^Faculty of Biology, Institute of Organismic and Molecular Evolution, Johannes Gutenberg University Mainz, Mainz, Germany; ^2^Neuroproteomics, Max-Delbrück Center for Molecular Medicine, Berlin, Germany; ^3^Department of Neuropsychiatry, Charité—Universitätsmedizin Berlin, Berlin, Germany; ^4^Charité—Universitätsmedizin Berlin, Virchow-Klinikum, Berlin-Brandenburger Centrum für Regenerative Therapien, Berlin, Germany; ^5^Klinik f. Anästhesiologie m.S. operative Intensivmedizin, Virchow Klinikum, Charité—Universitätsmedizin Berlin, Berlin, Germany; ^6^German Center for Neurodegenerative Diseases, Bonn, Germany; ^7^Department for Computational Molecular Biology, Max Planck Institute for Molecular Genetics, Berlin, Germany; ^8^Max-Planck-Institute for Molecular Genetics, Berlin, Germany; ^9^German Centre for Neurodegenerative Diseases, Berlin Institute of Health, Berlin, Germany; ^10^Centre for Clinical Brain Sciences, UK Dementia Research Institute, University of Edinburgh, Edinburgh, United Kingdom

**Keywords:** RNA-Seq, differential gene expression, Huntington's disease, disease markers, inflammation

## Abstract

Huntington's disease (HD) is an autosomal dominantly inherited neurodegenerative disorder caused by a trinucleotide repeat expansion in the *Huntingtin* gene. As disease-modifying therapies for HD are being developed, peripheral blood cells may be used to indicate disease progression and to monitor treatment response. In order to investigate whether gene expression changes can be found in the blood of individuals with HD that distinguish them from healthy controls, we performed transcriptome analysis by next-generation sequencing (RNA-seq). We detected a gene expression signature consistent with dysregulation of immune-related functions and inflammatory response in peripheral blood from HD cases vs. controls, including induction of the interferon response genes, *IFITM3, IFI6* and *IRF7*. Our results suggest that it is possible to detect gene expression changes in blood samples from individuals with HD, which may reflect the immune pathology associated with the disease.

## Introduction

Huntington's disease (HD) is an autosomal dominant neurodegenerative disease that commonly presents in mid-life with a triad of motor, cognitive, and psychiatric symptoms. HD is caused by a CAG trinucleotide repeat expansion in the first exon of the *Huntingtin* gene on chromosome 4 (1). Although the gene was identified more than 20 years ago, the pathophysiology of HD is still unclear. Huntingtin (HTT) is a ubiquitously expressed essential protein that is believed to acquire a toxic gain of function from the expanded polyglutamine stretch in the N-terminal part of the protein (2). Neurons are particularly vulnerable to mutant huntingtin (mHTT), but there is increasing evidence that the immune system is also affected. Microglia, the innate immune cells of the central nervous system (CNS), are activated even before the onset of symptoms in HD (3), and the degree of microglial activation correlates with disease severity (4). The cerebrospinal fluid and striatum of people with HD exhibit abnormal immune activation, suggesting that immune dysfunction plays a role in HD pathogenesis (5). Loss of Foxp1-mediated gene regulation may contribute to the immune dysfunction in HD (6). Notably, leukocytes in peripheral blood express mHTT, show migration deficits and are pathologically hyperactive (5, 7–9). The levels of mHTT in circulating monocytes and T cells are significantly associated with disease burden scores and caudate atrophy rates in individuals with HD (9).

The inflammatory changes detected in peripheral blood have been suggested to be shared between blood and brain in HD (10, 11). In fact, specific genomic profiles have also been identified in the blood of individuals with stroke, multiple sclerosis, and many other neurological diseases (12). Using two different microarray platforms, more than 300 mRNAs showed significantly altered expression in HD blood samples compared with controls (13). Expression of a subset of 12 genes clearly distinguished individuals with HD from healthy controls and correlated with disease progression (13). However, these findings were not confirmed in a follow-up study that identified the *Immediate Early Response 3* gene as a promising candidate (14). An increase in myeloperoxidase/white blood cell ratio was also detected in the blood of HD cases (15). Here, we have used next-generation sequencing to analyze differential RNA expression in blood samples from individuals with HD and age-matched controls from a single site in Germany in order to provide additional evidence for a blood signature of the disease.

## Results

Peripheral venous blood was collected from HD cases, including two pre-symptomatic HD mutation carriers and 9 symptomatic HD cases, and 8 age-matched controls without CNS disease (average age 55 and 58 years, respectively; *p* = 0.26 Student's *t*-test). The number of CAG repeats in the HD cases was between 41 and 43 (average = 42.0, stdev = 0.63). RNA was isolated and analyzed using deep sequencing of RNA transcripts (RNA-seq, see section Methods). The data were examined for differential gene expression, as well as for differential expression of individual exons and transcript isoforms.

### RNA-seq Gene Expression Analysis

**Figure 1:**
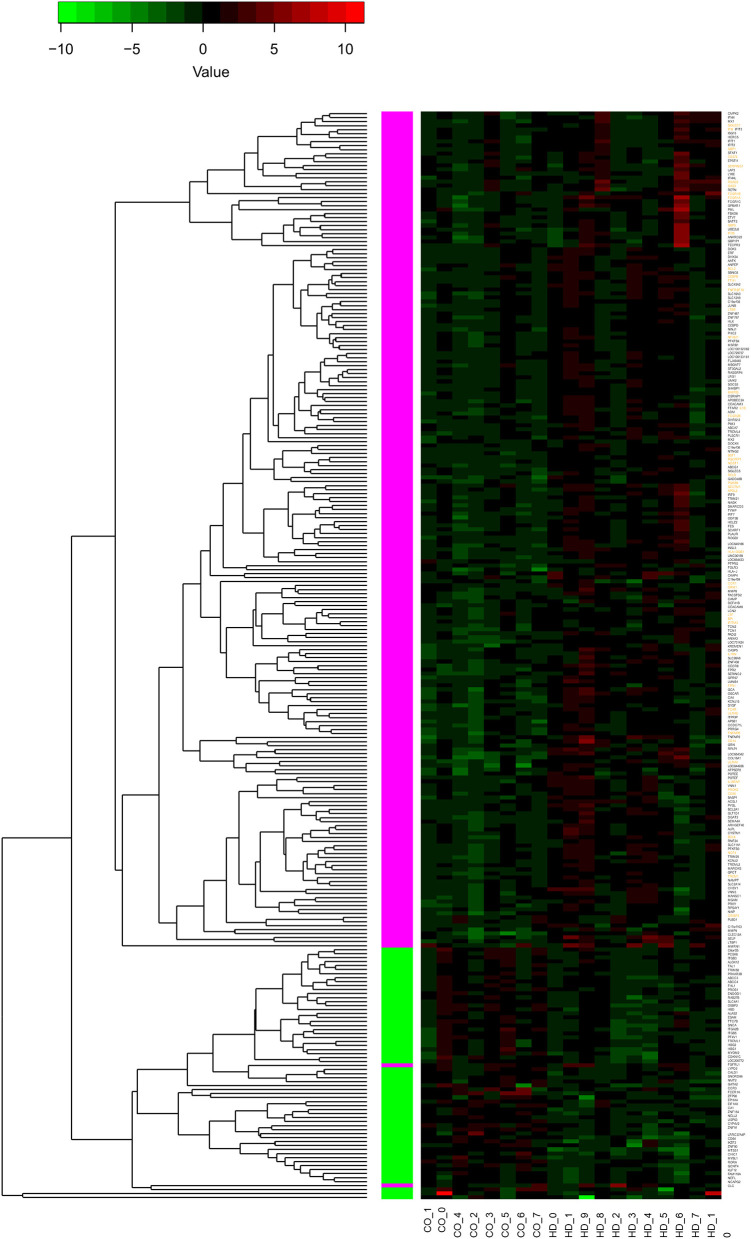
Differential expression of 274 genes. The heatmap represents the expression values of 274 genes with significant differential expression in peripheral blood of individuals with HD compared to controls (*q*-value < 0.05). The values are z-scores based on the log2 (FPKM) values, with 0 FPKM values set to 0.01, and modified to account for outliers following (16). Labels at the bottom indicate sample type: controls to the left (CO_), HD to the right (HD_). The dendrogram indicates results of hierarchically clustering the genes according to their expression values. Genes upregulated or downregulated in HD with respect to controls are indicated with magenta and green boxes, respectively. Gene labels in orange indicate genes with functions related to inflammation or immune response (see text for details); all of these genes are upregulated.

**Figure 2:**
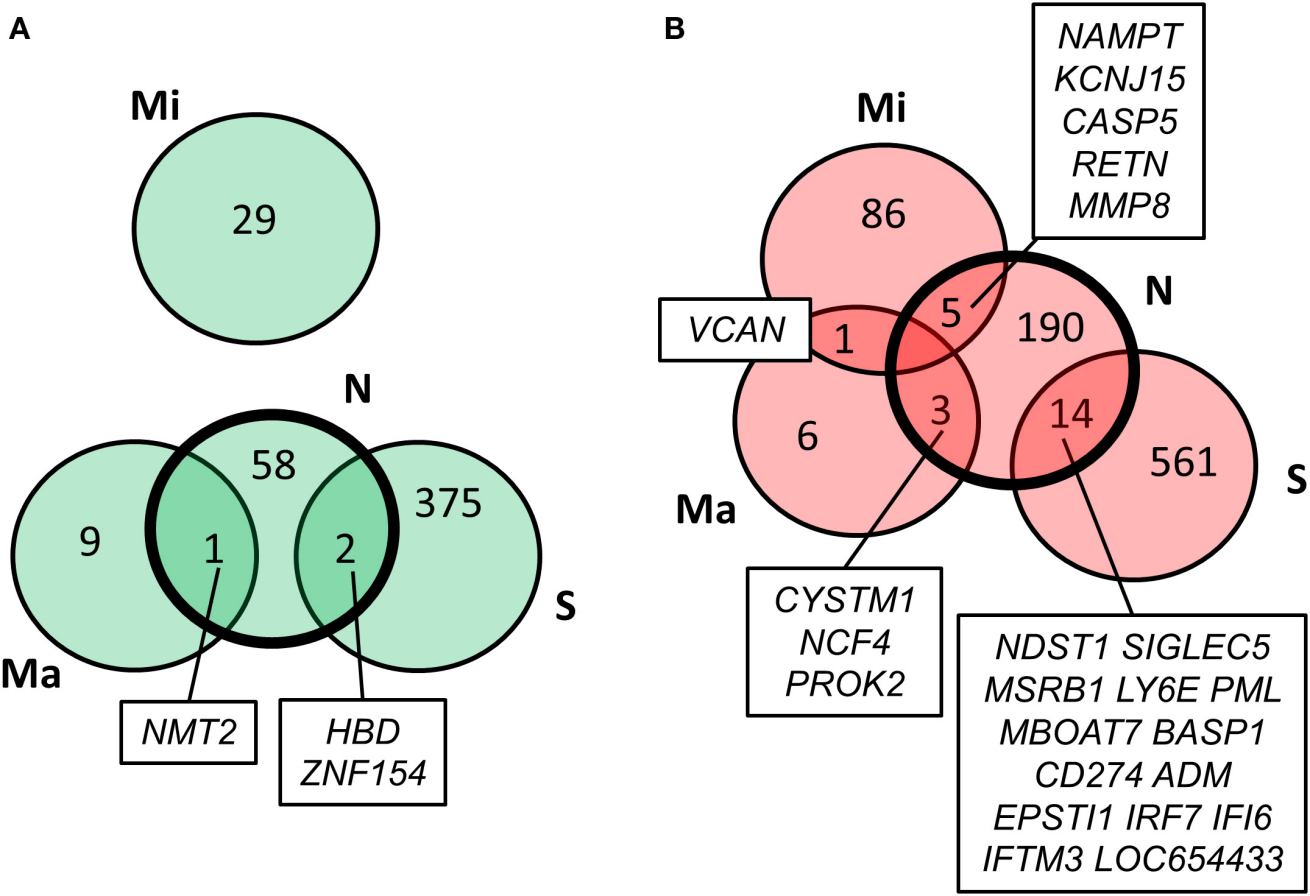
Comparison of our results with previous HD transcriptome analyses. Overlap of our set of dysregulated genes (N) with genes reported by Stopa et al. (22) (S), Mastrokolias et al. (19) (Ma), and Miller et al. (20) (Mi). **(A)** downregulated genes; **(B)** upregulated genes. Our set overlaps with the previous three datasets while the only overlap between the three previous datasets is the upregulation of VCAN in (19) and (20).

Values of expression were derived for transcripts (Supplementary Table 1; see section Methods). A total of 213 genes were found to be significantly upregulated, and 61 genes were significantly downregulated in HD cases vs. controls (*q*-value < 0.05; Figure 1). Gene Ontology (GO) enrichment analysis of these gene sets using all detected genes as background (23,604 genes) was performed using GOstat [using goa_human annotations; (17)]. Notably, immune-related functions were found in the set of upregulated genes. A total of 38 genes (545 in background) were related to immune response (*p*-value = 2e-35; Bonferroni corrected hereafter), and 20 genes (270 in background) were related to inflammatory response (*p*-value = 3e-8) with some overlap between the sets. In contrast, the only GO term found to be enriched in the downregulated genes was hemoglobin complex (associated to three genes *HBD, HBG1* and *HBG2* vs. 11 in background; *p*-value = 0.004). In Table 1, we summarize how our dataset of dysregulated genes in HD peripheral blood compares to previous studies using blood and CNS tissue from HD cases (10, 11, 13, 14, 18–22). Importantly, the expression of two interferon-induced genes, *IFITM3 and IFI6* (*G1P3*), as well as *CD274* (*PDL1*) and *NDST1* was upregulated in both peripheral blood (our study) and choroid plexus (22) of HD cases (Figure 2). The upregulation of *NCF4* and *PROK2* in HD peripheral blood cells was shared between our study and Mastrokolias et al. (19) (Figure 2). Interestingly, we found more overlap between studies for upregulated genes than for downregulated genes (Figure 2).

**Table 1:** Comparison of our dataset of dysregulated genes in HD with gene sets from relevant studies.

**Study**	**Sample**	**Transcriptome analysis**	**Dataset/figure and table numbers in referred publication**	**Overlap to our set of 213 up- and 61 down-regulated genes**
Borovecki et al. (13) PNAS	HD blood	Oligonucleotide microarrays	322 differentially expressed genes/Figure 1	Gene list not available
			12 upregulated genes/Figure 2A	No overlap
Runne et al. (14) PNAS	HD blood	Oligonucleotide microarrays	19 upregulated genes/Table 3	1 gene (*IL1B*)
Chang et al. (18) PLoS ONE	HD blood	RT-PCR	4 downregulated genes	No overlap
Hensman Moss et al. (11)	HD blood	RNA-seq	Upregulated and downregulated pathways	Comparison not possible
Mastrokolias et al. (19) Eur J Hum Genet	HD blood	DeepSAGE	99 up- and 68 down-regulated genes	Gene list not available
			10 up- and 10 down-regulated genes/Table 2	3 upregulated genes (*CYSTM1, NCF4, PROK2*) and 1 down-regulated gene (NMT2)
			5 validated upregulated genes	3 genes (*ANXA3, CYSTM1, PROK2*)
Miller et al. (20) Hum Mol Genet	HD monocyte cultures	RNA-seq	101 up- and 29 down-regulated genes/dataset S2 (FDR < 0.05)	5 upregulated genes (*NAMPT, KCNJ15, CASP5, RETN, MMP8*)
Labadorf and Myers (21) PLoS ONE	HD brain	RNA-seq	Alternative splice variants	Comparison not possible
Mina et al. (10) Orphanet J Rare Dis	HD brain and blood	Oligonucleotide microarrays (brain) and RNA-seq (blood)	Gene modules	Comparison not possible
Stopa et al. (22) Fluids Barriers CNS	HD choroid plexus	Oligonucleotide microarrays	Supplementary Table 2: 584 up- and 377 down-regulated genes (*p*-value < 0.01)	14 up- and 2 down-regulated genes (*HBD, ZNF154*)

### RNA-seq Exon Expression Analysis

One of the strengths of the RNA-seq data is that it allows for the analysis of differential expression at the level of independent exons. In order to identify exons that are differentially expressed in HD cases vs. controls, we evaluated the expression of a total of 196,877 exons from 25,897 transcripts. Then, we selected differentially expressed exons having more than 50 reads in control samples, and at least five HD cases with z-scores > 4 (upregulation) or at least five HD cases with z-scores < −4 (downregulation). According to this selection procedure, we identified 38 upregulated exons and 75 downregulated exons from 107 transcripts (Supplementary Table 2). Using the same selection procedure on 1,000 randomized datasets, we estimated a *p*-value of 0.053 for the selection of 113 or more exons. Consistently, we did not find significant differential expression of gene transcript isoforms between HD cases and controls [using MISO (23), see section Methods for details].

## Discussion

The study of gene expression in the peripheral blood of individuals with HD has produced controversial results. The first study in peripheral blood using oligonucleotide microarrays found 322 differentially expressed (mostly upregulated) genes in five pre-symptomatic and 12 manifest HD cases vs. 14 controls (13). This study presented a selection of 12 marker genes upregulated in the blood of HD cases, of which seven were also upregulated in post-mortem HD brain samples. However, a subsequent study failed to replicate these findings and detected only a modest increase in the expression of the immediate early gene *IER3* in the blood of 12 HD cases vs. 10 controls (14). A quantitative PCR study in peripheral blood leukocytes from four pre-symptomatic and 16 manifest HD cases vs. 20 controls detected four downregulated genes involved in metabolism and oxidative stress (18), none of which were regulated in our study. However, we observed increased expression of *IL1B* and genes involved in TNF signaling similar to the findings of Runne et al. (14) in HD peripheral blood cells, suggesting a proinflammatory profile. More recently, RNA-seq was used to study gene expression in peripheral blood from pre-manifest and manifest HD mutation carriers. Interestingly, Miller et al. (20) identified TNF and IL1B as the transcriptional regulators with the highest target gene overlap with changes in the HD monocyte transcriptome when comparing 30 HD cases with 33 controls. Five upregulated genes (*NAMPT, KCNJ15, CASP5, RETN, MMP8*) in Miller et al. (20) were shared with our data set. Immune pathways were also enriched in a large DeepSAGE analysis of peripheral blood from 27 pre-manifest and 64 manifest HD mutation carriers vs. 33 controls (19). This important study identified the five genes *ZNF238, AQP9, CYSTM1, ANXA3*, and *PROK2* as HD biomarkers. Notably, *ANXA3* (encoding Annexin A 3) and the circadian clock-controlled gene *PROK2* (encoding Prokineticin 2) were also induced in HD leukocytes in our study. Likewise, both studies observed increased expression of the *NCF4* gene in HD leukocytes, which encodes the p40phox subunit of the phagocyte NADPH oxidase that is crucial for the innate immune response. Hensman Moss et al. (11) examined peripheral blood from two large cohorts of 72 pre-manifest and 119 manifest HD cases and 49 controls by RNA-seq, which revealed no differential expression of individual transcripts in HD but a significant upregulation of immune-related pathways in myeloid cells that replicated those in the HD brain. A good overlap between blood and brain signatures in HD was also observed in three transcriptomic studies of post-mortem brain tissue demonstrating pronounced changes for immune-related genes (10, 21, 24). When transcriptome-wide Affymetrix microarrays were used to compare gene expression in choroid plexus from three grade IV HD cases vs. six controls (22), 14 upregulated genes and 2 downregulated genes matched the differentially expressed genes that we detected in HD leukocytes in this study. Most notably, the interferon response genes, *IFITM3* (encoding Interferon-induced transmembrane protein 3), *IFI6* (encoding Interferon alpha-inducible protein 6), *IRF7* (Interferon regulatory factor 7), *CD274* (encoding Programmed death-ligand 1), and *NDST1* (encoding N-deacetylase-N-sulfotransferase-1) were induced in both peripheral blood and choroid plexus in HD. IFITM3 has recently been identified as a modulator of γ-secretase activity that is associated with aging and Alzheimer's disease (25). IFITM3 was also induced in activated microglia in response to β-amyloid (26). The *IRF7* gene has been associated with frontotemporal lobar degeneration (27). Finally, enrichment of genes related to immune response was detected in a study that assessed longitudinal transcriptomic dysregulation in the peripheral blood of transgenic monkeys with genomic integration of pathogenic fragments of the human *HTT* gene (28).

In sum, our results suggest a peripheral blood signature of HD that involves immune-related genes, in particular the interferome. Further research and validation in large cohorts with prospective longitudinal assessments by RNA-seq should contribute to refine the set of genes that mirror HD in blood.

## Materials and Methods

### Ethics Approval

All human experiments were performed in accordance with the Declaration of Helsinki, and were approved by the regulatory authorities (Ethikkommission der Charité Campus Mitte, Berlin, Germany). All study participants gave written informed consent.

### Study Participants

Subjects were genetically diagnosed with HD and examined by experienced neurologists. Disease stage was determined based on the UHDRS motor score and total functional capacity (29), and the Folstein mini-mental state examination. Control subjects were free of CNS disease. None of the study participants suffered from inflammatory or infectious conditions.

### RNA Extraction From Blood Samples

Peripheral venous blood was collected (3 × 10 ml) from HD patients and controls into tubes containing EDTA in the mornings of 7^th^−10^th^ September 2009. RNA was immediately extracted using the LeukoLOCKTM total RNA isolation system according to the manufacturer's protocol (Ambion, TX, USA). All samples were analyzed separately and not pooled. Briefly, each 10 ml blood sample was passed through a LeukoLOCKTM filter, followed by flushing the filter with 3 ml phosphate-buffered saline (PBS), and subsequently with 3 ml RNAlater®. After removing the residual RNAlater® from the LeukoLOCKTM filter, the filter was flushed with 2.5 ml pH-adjusted Lysis/Binding solution. The lysate was collected into 15 ml tubes containing 2.5 ml nuclease-free water, followed by addition of 25 μl proteinase K and shaking on a roto-rack for 5 min. For RNA isolation, 50 μl RNA-binding beads and 2.5 ml 100% isopropanol were added, incubated at room temperature for 5 min and centrifuged at 2,000 × g for 3 min. Supernatant was discarded. RNA-binding beads were washed with 1.2 ml wash solution, and transferred into a 1.5 ml reaction tube. The samples were centrifuged at 16,000 × g for 30 s, and the supernatant was discarded. The RNA-binding beads were washed twice with 750 μl wash solution 2/3. The pellet was air-dried. RNA was removed from the RNA-binding beads using 100 μl elution solution. After centrifugation at 16,000 × g for 2 min, the supernatant containing the RNA was transferred to a new nuclease-free reaction tube, and stored at −80°C. The quality and yield of extracted RNA were analyzed on a total eukaryote RNA nano chip using the 2,100 Bioanalyzer (Agilent Technologies, Germany), and by UV spectrography using Nanodrop 1,000 (Thermo Scientific, Germany).

### RNA-seq Profiling

Poly(A)+ selected RNA libraries were prepared according to a previously published protocol while preserving the strand information (30).

### RNA-seq Analysis

#### Sequencing and Mapping

Sequencing was carried out on an Illumina Genome Analyzer II System with 2 × 51 cycles. Raw paired-end reads in fastq format were mapped to human reference genome (hg19) using TopHat (v2.0.9) and Bowtie2 (v2.1.0) (31, 32), with the following parameters for TopHat: –min-anchor-length = 8, –splice-mismatches = 1, –min-intron-length = 40, –max-intron-length = 1,000,000, –mate-inner-dist = 150, –solexa 1.3-quals, –coverage-search. Only reads with MAPQ ≥ 1 were considered for expression analysis.

#### RNA-seq Data Analysis

Coordinates for genes and exons were downloaded from the Ensembl Genes 53 database using BioMart tool with Homo sapiens genes (NCBI36) dataset. Only the intervals on chromosomes 1–22, X, Y, M with merged overlapping exons belonging to the same gene were used to calculate exon hits. Any read overlapping with at least 1 bp to an exon was counted. A custom script based on the pysam module [http://pysam.readthedocs.org; (33)] was used to count the number of sense- and antisense reads that overlap each exon interval by at least one base. The strand-specific RNA-Seq protocol used herein allowed us to include only reads belonging to the original orientation of transcription orientation for the calculation of exons. Gene exon RPKM values were calculated according to exon hits and normalized against the library size (total MAPQ ≥ 1 reads) and the merged length of the coding sequence of each gene.

Mapped reads were subjected to differential gene analysis using Cufflinks (v2.1.1) using default parameters and UCSC hg19 reference gene annotation (34).

### Exon Expression Analysis

For each sample and for each gene exon, we counted the number of reads with an alignment overlapping the exon location. We did not count reads with an alignment overlapping multiple exons. We then scaled the counts *c*_*i*_ for each exon *i* in a transcript as *s*_*i*_ = *c*_*i*_*-*μ*/*σ, where μ is the mean of the *c*_*i*_ values in the transcript and σ is their standard deviation. Then, we computed a z-score for each exon in each of the patients, comparing the (scaled) expression of a given exon in a patient and the reference distribution of (scaled) expression for this exon in the control samples. Randomized datasets were generated by random permutations of sample labels.

### Alternative Expression Analysis

We used MISO (23) version (0.5.3) to obtain transcript ratios (PSI values) from hg19 annotation. Afterwards, in order to detect possible genes with significantly altered transcript isoforms we used the rasp package (https://rdrr.io/github/isglobal-brge/rasp/f/inst/doc/rasp.pdf), a distance-based non-parametric multivariate approach as described in Gonzalez-Porta et al. (35) and Iglewicz and Hoaglin (16). We did not find isoforms significantly differentially expressed between the two groups.

## Data Availability Statement

The datasets generated in this study can be found in online repositories. The names of the repository/repositories and Accession Number(s) can be found below: https://www.ncbi.nlm.nih.gov/geo/, GSE61405.

## Ethics Statement

The studies involving human participants were reviewed and approved by Ethikkommission der Charité Campus Mitte, Berlin, Germany. The patients/participants provided their written informed consent to participate in this study.

## Author Contributions

JP and EW designed the study. JP, ES, and KM collected the clinical samples. VA, AS, and WN processed the samples for transcriptomics. KM, NM, AG-L, TA, JR, MH, EM, J-FF, and MA-N analyzed the data. MA-N and JP wrote the main manuscript. All authors reviewed the manuscript.

## Conflict of Interest

The authors declare that the research was conducted in the absence of any commercial or financial relationships that could be construed as a potential conflict of interest.

## Supplementary Material

Supplementary Table 1 Differentially regulated genes.

Supplementary Table 2 Differentially expressed exons.
